# Translationally Controlled Tumor Protein Stimulates Dopamine Release from PC12 Cells via Ca^2+^-Independent Phospholipase A_2_ Pathways

**DOI:** 10.3390/ijms17101774

**Published:** 2016-10-24

**Authors:** Jihui Seo, Jeehye Maeng, Hwa-Jung Kim

**Affiliations:** College of Pharmacy, Graduate School of Pharmaceutical Sciences, Ewha Womans University, Seoul 120-750, Korea; hui7505@hanmail.net (J.S.); jhmaeng@ewha.ac.kr (J.M.)

**Keywords:** translationally controlled tumor protein, histamine releasing factor, dopamine release, Ca^2+^-independent pathways, phospholipase A_2_, PC12 neuronal cells

## Abstract

The translationally controlled tumor protein (TCTP), initially identified as a tumor- and growth-related protein, is also known as a histamine-releasing factor (HRF). TCTP is widely distributed in the neuronal systems, but its function is largely uncharacterized. Here, we report a novel function of TCTP in the neurotransmitter release from a neurosecretory, pheochromocytoma (PC12) cells. Treatment with recombinant TCTP (rTCTP) enhanced both basal and depolarization (50 mM KCl)-evoked [^3^H]dopamine release in concentration- and time-dependent manners. Interestingly, even though rTCTP induced the increase in intracellular calcium levels ([Ca^2+^]_i_), the rTCTP-driven effect on dopamine release was mediated by a Ca^2+^-independent pathway, as evidenced by the fact that Ca^2+^-modulating agents such as Ca^2+^ chelators and a voltage-gated L-type Ca^2+^-channel blocker did not produce any changes in rTCTP-evoked dopamine release. In a study to investigate the involvement of phospholipase A_2_ (PLA_2_) in rTCTP-induced dopamine release, the inhibitor for Ca^2+^-independent PLA_2_ (iPLA_2_) produced a significant inhibitory effect on rTCTP-induced dopamine release, whereas this release was not significantly inhibited by Ca^2+^-dependent cytosolic PLA_2_ (cPLA_2_) and secretory PLA_2_ (sPLA_2_) inhibitors. We found that rTCTP-induced dopamine release from neuronal PC12 cells was modulated by a Ca^2+^-independent mechanism that involved PLA_2_ in the process, suggesting the regulatory role of TCTP in the neuronal functions.

## 1. Introduction

The translationally controlled tumor protein (TCTP) was initially reported as a tumor-associated and growth-related protein (reviewed in [1]). Its homologs from mammalian species were reported to show extensive similarities, approximately 95% among rats and mice (P21), and humans (P23) [2,3]. In addition, TCTP was characterized as an extrinsic histamine releasing factor (HRF) because of its cytokine-like activities [4]. TCTP has been attracted an increasing number of researchers’ attention because of its diverse biological functions and potentially important roles in medically relevant processes based on the fact that TCTP is ubiquitously expressed in a variety of different tissues and cell types, and its levels are highly regulated in response to various extracellular stimuli at both transcriptional and translational levels (reviewed in [1]). TCTP has been recognized as a multifunctional protein with diverse physiological and pathological roles in cell-cycle progression, proliferation, oncogenic tumorigenesis, stress response, gene regulation, cell death, and protection against cell death induced by heat shock stress, or oxidative stress. Numerous biological studies regarding its broad spectrum of functional importance have been presented [5], but its detailed role of TCTP especially in the nervous system remains largely elusive.

Mounting evidence has indicated the potential for the regulatory roles of TCTP in the brain functions. TCTP mRNA is expressed in many areas of the adult human brain [6], and significantly decreased TCTP expression was observed in the temporal cortex of patients with Alzheimer’s disease and Down’s syndrome [7] and in the schizophrenia hippocampus [8]. More recently, transcripts that encode TCTP were found to be enriched in the axonal compartment of embryonic and adult neurons [9,10,11,12], suggesting that TCTP plays an important but still unknown role in neuronal systems.

Here, motivated by the suggestive implications of TCTP in neurons and its localization in neuronal systems, we investigated the role of TCTP in the neurotransmitter release from a neurosecretory cell type, pheochromocytoma (PC12) cells. Our present study reports that TCTP enhances both basal and depolarization-evoked [^3^H]dopamine release and that the TCTP-triggered dopamine release from PC12 cells is mediated by Ca^2+^-independent, phospholipase A_2_ (PLA_2_)-involved pathways.

## 2. Results

### 2.1. Expression of Endogenous Translationally Controlled Tumor Protein (TCTP) in PC12 Cells and Rat Brain Tissues

**Figure 1:**
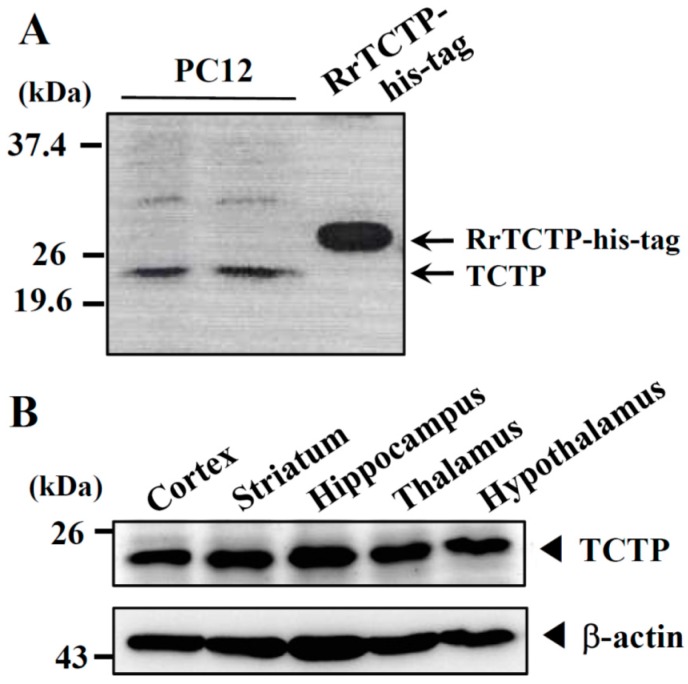
Endogenous translationally controlled tumor protein (TCTP) expression in PC12 cells and rat brain. Proteins extracted from PC12 cells (3 × 10^6^ cells, about 40 µg; (**A**)), recombinant RrTCTP-his-tag (10 µg; (**A**)); and rat brain tissues (indicated regions, 40 µg; (**B**)) were analyzed by immunoblotting using murine anti-TCTP antibody.

To examine the endogenous expression of TCTP in PC12 cells, PC12 cell homogenates were analyzed by Western blotting using a polyclonal antibody against murine TCTP. We found that the rat-originated PC12 cells were found to express TCTP with the expected size of approximately 21 kDa (Figure 1A). The ubiquitous expression of TCTP was also confirmed in the brain regions in which dopaminergic neuronal pathways are known to be present, such as the cortex, striatum, hippocampus, thalamus, and hypothalamus (Figure 1B).

### 2.2. Stimulatory Effect of TCTP on Basal and Depolarization-Induced Release of Dopamine from PC12 Cells

**Figure 2:**
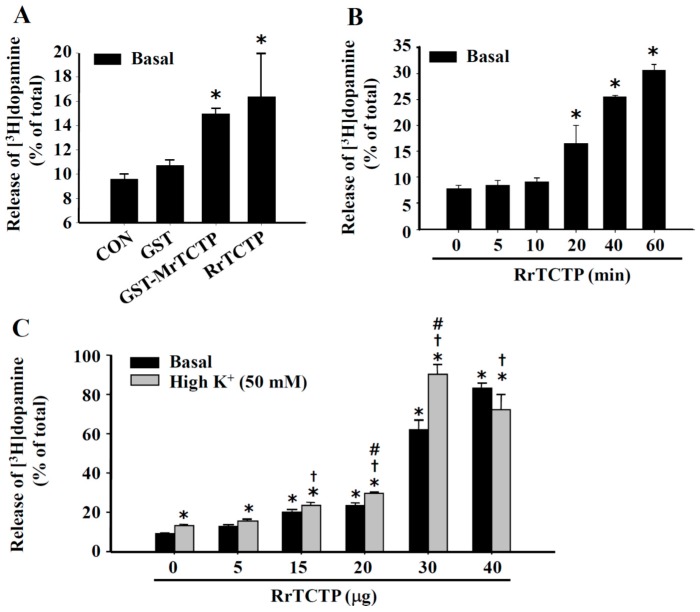
TCTP stimulates both basal spontaneous and depolarization (50 mM KCl)-induced release of dopamine from PC12 cells. PC12 cells preloaded with [^3^H]dopamine were washed and incubated at 37 °C with rat recombinant TCTP (RrTCTP) (15 µg/mL, (**A**,**B**); 5–40 µg/mL, (**C**)), GST-tagged mouse recombinant TCTP (GST-MrTCTP) (50 µg/mL, (**A**)) or GST (50 µg/mL, (**A**)) for 20 min (**A**,**C**) or indicated time periods (**B**), under basal (5 mM KCl) or high K^+^ (50 mM KCl) KR buffer conditions. The amount of [^3^H]dopamine released into the culture supernatant was significantly increased by both RrTCTP and GST-MrTCTP, but not by GST alone (**A**). The rTCTP-induced stimulation of dopamine release was time- (**B**) and dose-dependent (**C**). * *p* < 0.05 relative to basal control release; † *p* < 0.05 relative to high K^+^ control release; # *p* < 0.05 relative to basal release.

Recombinant TCTP proteins including rat recombinant TCTP (RrTCTP) and glutathione *S*-transferase (GST)-tagged mouse recombinant TCTP (GST-MrTCTP) were expressed in *Escherichia coli* and purified as described in “Materials and Methods”. To test whether TCTP could induce both basal and depolarization-induced dopamine release from neurosecretory PC12 cells, the cells were treated with recombinant TCTPs (rTCTPs) for 20 min. Both RrTCTP and GST-MrTCTP enhanced the 20-min dopamine release from PC12 cells under basal (5 mM KCl and Ca^2+^-containing) KR buffer conditions. RrTCTP increased dopamine release by 65% and GST-MrTCTP increased dopamine release by 50% compared with in the control (CON) groups, whereas GST (50 μg/mL) alone produced no effect (Figure 2A). The rTCTP-induced stimulation of basal dopamine release was time-dependent; RrTCTP (15 μg/mL) stimulated the dopamine release at incubation times of 20–60 min (Figure 2B). RrTCTP significantly enhanced both the spontaneous (basal) and the high K^+^ (50 mM KCl)-stimulated release of dopamine, at 15–40 μg/mL RrTCTP in a concentration-dependent manner (Figure 2C).

### 2.3. Regulation of Intracellular Ca^2+^ Levels by TCTP in PC12 Cells

**Figure 3:**
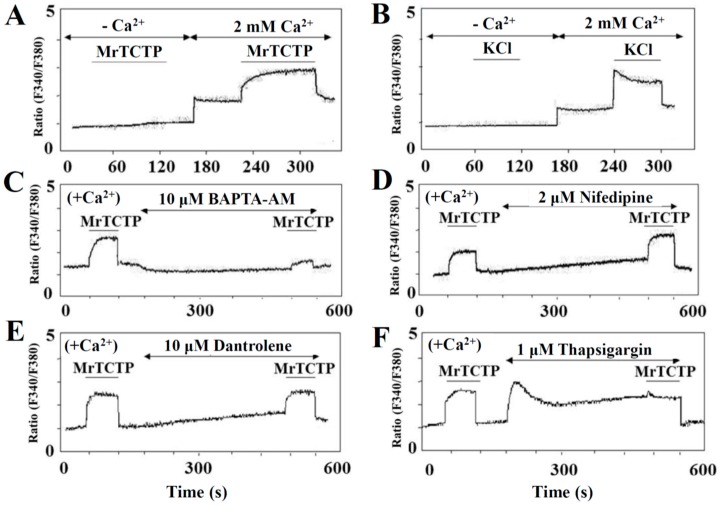
TCTP increases intracellular Ca^2+^ concentration. PC12 cells were loaded with 5 µM Fura-2-AM (acetoxymethyl) for 30 min and then washed multiple times. The fluorescence experiments were carried out at room temperature in the Ca^2+^ (2 mM CaCl_2_)-containing buffer (+Ca^2+^), or the Ca^2+^-free buffer supplemented with 0.5 mM ethylene glycol-bis(β-aminoethyl ether)-*N*,*N*,*N*′,*N*′-tetraacetic acid (EGTA) (−Ca^2+^). Cells were treated for the indicated times with 50 µg/mL MrTCTP (**A**) or 50 mM KCl (**B**). In some experiments, cells were treated with MrTCTP after pretreatment for 5 min with 10 µM BAPTA-AM (**C**); 2 µM niffedipine (**D**); and 10 µM dantrolene (**E**); or 1 µM thapsigargin (**F**).

It is well-known that the neurotransmitter release stimulated by depolarization is Ca^2+^-dependent exocytotic release, but the spontaneous basal neurotransmitter release is not. To characterize the TCTP-induced stimulation of dopamine release from PC12 cells, we first investigated whether rTCTP triggered an increase in intracellular cytosolic Ca^2+^-concentration ([Ca^2+^]_i_). Fura-2-acetoxymethyl ester (AM), a widely used membrane permeable Ca^2+^ indicator, is known to be hydrolyzed by intracellular esterases, and then trapped within the cytoplasm. Fura-2-AM-loaded PC12 cells were treated with MrTCTP or KCl in the Ca^2+^-free or Ca^2+^-containing KR buffer. MrTCTP evoked a gradual and sustained increase in [Ca^2+^]_i_ in the Ca^2+^-containing buffer condition but not in the absence of extracellular Ca^2+^ (Figure 3A), similar to the effects on [Ca^2+^]_i_ with 50 mM KCl; these reactions mimicked those in the highly depolarized condition except that KCl produced a rapid rise in [Ca^2+^]_i_ (Figure 3B). Removal of cytosolic Ca^2+^ by BAPTA-AM, a cell-permeant Ca^2+^ chelator, reduced the basal level of [Ca^2+^]_i_ and abolished the rTCTP-induced [Ca^2+^]_i_ rise in the Ca^2+^-containing buffer condition (Figure 3C). It has been shown that, in PC12 cells, membrane depolarization by KCl results in an influx of Ca^2+^ through depolarization-induced activation of voltage-sensitive Ca^2+^ channels, thereby triggering exocytosis [13,14]. The Ca^2+^ current evoked by rTCTP was not affected by treatment with an L-type Ca^2+^ channel inhibitor, nifedipine (2 µM), as shown in Figure 3D; meanwhile Ca^2+^ current evoked by KCl was inhibited (data not shown). This finding suggests that unlike KCl, voltage-gated L-type Ca^2+^ channels are not involved in MrTCTP-induced Ca^2+^ current.

Next, it was further investigated whether the rTCTP-induced [Ca^2+^]_i_ increase was due to the mobilization of Ca^2+^ from intracellular stores by utilizing dantrolene, which blocks Ca^2+^ release from the endoplasmic reticulum (ER). The rTCTP-evoked cytosolic Ca^2+^ current was also not affected by dantrolene (10 µM), as shown in Figure 3E. Our data indicate that the rTCTP-induced increase in [Ca^2+^]_i_ was due to a Ca^2+^ influx from an extracellular source rather than via voltage-gated L-type Ca^2+^ channels, and that rTCTP does not mobilize Ca^2+^ from intracellular stores. Thapsigargin, an inhibitor of Ca^2+^-ATPase in the membranes of ER Ca^2+^ stores, is generally used to inhibit refilling of Ca^2+^ stores and subsequently depleting Ca^2+^ from Ca^2+^ stores in various cells [15,16], including PC12 cells. When PC12 cells were exposed to 1 µM thapsigargin in a Ca^2+^-containing buffer, it caused a sustained increase in [Ca^2+^]_i_, confirming that intracellular Ca^2+^ stores are releasable under our experimental conditions. When 50 µg MrTCTP were added in the medium that contained both Ca^2+^ and thapsigargin, further rise in [Ca^2+^]_i_ was not produced by rTCTP (Figure 3F).

### 2.4. Ca^2+^-Independent Release of Dopamine by TCTP from PC12 Cells

**Figure 4:**
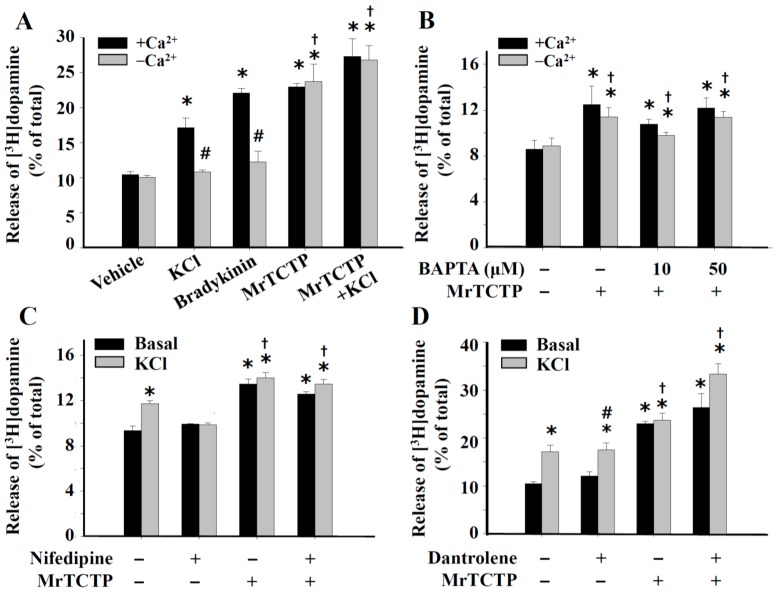
TCTP induced dopamine release from PC12 cells via Ca^2+^-independent pathways. [^3^H]Dopamine-preloaded PC12 cells were washed and treated in Ca^2+^-containing buffer (+Ca^2+^) or Ca^2+^-free buffer (−Ca^2+^) with 50 mM KCl, 1 µM bradykinin, and 50 µg/mL MrTCTP (**A**); [^3^H]Dopamine-preloaded PC12 cells were washed, pretreated for 20 min at 37 °C with 10 or 50 µM BAPTA-AM (**B**); 2 μM nifedipine (**C**); or 10 μM dantrolene (**D**); and then treated for 20 min with 50 µg/mL MrTCTP with the continued presence of above agent where indicated, in the basal and the high K^+^ (50 mM KCl) buffers. [^3^H]Dopamine release into media is expressed as relative to total [^3^H]dopamine loaded. Data are presented as Mean ± S.E.M. (standard error of mean) * *p* < 0.05 relative to control basal release; † *p* < 0.05 relative to control high K^+^ release; # *p* < 0.05 relative to respective basal release.

To investigate whether the rTCTP-evoked dopamine release was Ca^2+^-dependent and the [Ca^2+^]_i_ changes induced by rTCTP played a role in the rTCTP-induced enhancement of dopamine release, effects of Ca^2+^ chelators that removed extracellular and intracellular Ca^2+^ sources, and Ca^2+^ inhibitors (nifedipine or dantrolene) on dopamine release from PC12 cells were examined. Unexpectedly, rTCTP stimulated dopamine release even in the absence of extracellular Ca^2+^ by a similar extent to that in the presence of extracellular Ca^2+^. In contrast, both bradykinin (1 µM) and KCl (50 mM) stimulated dopamine release only in the Ca^2+^-containing media, and did not induce dopamine release in the absence of extracellular Ca^2+^ (Ca^2+^-free media + EDTA (ethylenediaminetetraacetic acid)) (Figure 4A). The Ca^2+^-independence of the rTCTP-evoked dopamine release was further confirmed by the observations that BAPTA-AM (10 and 50 µM), nifedipine (2 µM) and dantrolene (10 µM) did not significantly inhibit rTCTP-evoked dopamine release either in the presence or in the absence of extracellular Ca^2+^ (Figure 4B–D), although some of the agents blocked rTCTP-evoked intracellular Ca^2+^ peak in a Ca^2+^-containing buffer (Figure 3). These data indicate that even though rTCTP induced an influx of Ca^2+^, the rTCTP-induced enhancement of dopamine release was largely regulated by Ca^2+^-independent processes, and that [Ca^2+^]_i_ increased by rTCTP played only a minor role if any in the rTCTP-evoked dopamine release from PC12 cells.

### 2.5. Involvement of Ca^2+^-Independent Phospholipase A_2_ (PLA_2_) in TCTP-Induced Dopamine Release from PC12 Cells

It has been shown that a polypeptide composed of 33 amino acids—pardaxin—stimulates exocytosis in the neuronal systems by both Ca^2+^-dependent and Ca^2+^-independent mechanisms [17,18]. A direct relationship was proposed between pardaxin-induced dopamine release and the arachidonic acid cascades. Arachidonic acid, a major fatty acid produced by Ca^2+^-dependent or Ca^2+^-independent activation of PLA_2_, serves as a precursor of eicosanoids that mediate diverse physiological and pathophysiological processes in brain and other tissues [19,20]. 

**Figure 5:**
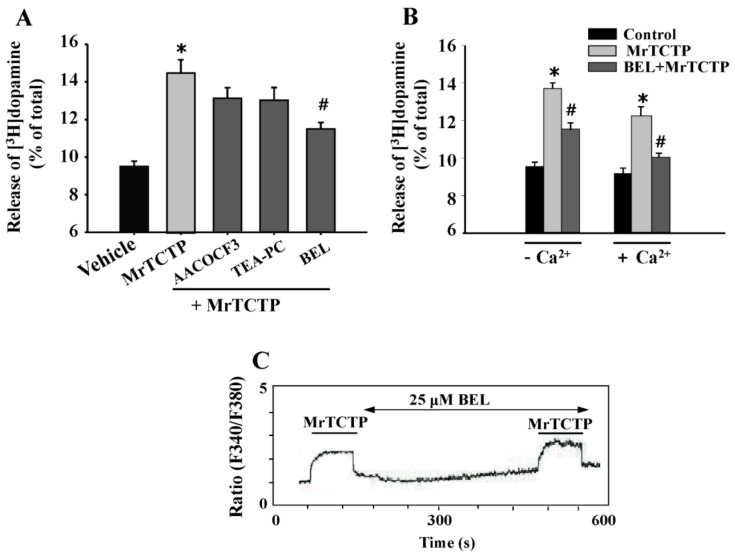
The effects of phospholipase A_2_ (PLA_2_) inhibitors on TCTP-induced dopamine release from PC12 cells. (**A**,**B**) PC12 cells were pre-incubated for 20 min at 37 °C with 25 µM AACOCF_3_, 10 µM TEA-PC, or 25 µM BEL, and then treated with 50 µg/mL MrTCTP for an additional 20 min in Ca^2+^-containing (+Ca^2+^), or Ca^2+^-free (−Ca^2+^) buffer. The amount of [^3^H]dopamine released into the culture supernatant was measured. * *p* < 0.05 relative to control release; # *p* < 0.05 relative to MrTCTP-treated release; (**C**) the fluorescence experiments were carried out at room temperature in the Ca^2+^ (2 mM CaCl_2_)-containing buffer. PC12 cells were loaded with 5 µM Fura-2-AM for 30 min and then washed multiple times. The cells were pretreated with 25 µM BEL for 5 min, and then exposed to 50 µg/mL MrTCTP.

To investigate whether the PLA_2_ signaling is also associated as a mechanism involved in the TCTP-induced dopamine release in PC12 cells, several inhibitors of PLA_2_ isoforms were used. PC12 cells were pre-incubated for 20 min with 25 µM arachidonyl trifluoromethyl ketone (AACOCF_3_) (a cytosolic PLA_2_ (cPLA_2_) inhibitor), 10 µM 1-palmitylthio-2-palmitoylamino-1,2-dideoxy-sn-glycero-3-phosphorylcholine (TEA-PC) (a secretory PLA_2_ (sPLA_2_) inhibitor), or 25 µM bromoenol lactone (BEL) (a Ca^2+^-independent PLA_2_ (iPLA_2_) inhibitor), and thereafter treated with 50 µg/mL MrTCTP for an additional 20 min in a Ca^2+^-containing medium. As shown in Figure 5A, TEA-PC and AACOCF3 produced no significant changes, but BEL significantly inhibited the rTCTP-induced dopamine release (Figure 5A), indicating the possibility of iPLA_2_ involvement in rTCTP-evoked dopamine release.

Among the three major PLA_2_ families, only iPLA_2_ can be activated under Ca^2+^-free conditions [21,22]. It has been reported that even though iPLA_2_ does not require Ca^2+^ for activity, it may be modulated by Ca^2+^ or Ca^2+^-dependent factors in cells [23], and it can regulate store-operated Ca^2+^ entry in rat cerebellar astrocytes [24]. Treatment with BEL (25 µM) significantly inhibited the release of dopamine induced by MrTCTP not only in Ca^2+^-containing buffer but also in Ca^2+^-free buffer (Figure 5B). However, BEL (25 µM) did not affect MrTCTP-evoked intracellular Ca^2+^ peak in a Ca^2+^-containing buffer (Figure 5C). These findings further confirm that iPLA_2_ is involved in the rTCTP-induced dopamine release from PC12 cells in a Ca^2+^-independent fashion.

## 3. Discussion

In the present study, we investigated the novel function of TCTP in dopamine release, focusing on its Ca^2*+*^-independent regulation of neurotransmitter release in rat pheochromocytoma PC12 cells in which endogenous TCTP protein expression is detected (Figure 1). The PC12 cells have been widely used and well characterized in in vitro models for studying neuronal function and signaling processes [25], since it was reported that dopamine, norepinephrine, ATP, and various proteins were stored in secretory vesicles, and chromaffin granules of undifferentiated PC12 cells [14].

TCTP is known to be involved in a great variety of physiological processes such as cellular proliferation and development, microtubule stabilization, protein synthesis, cell cycle progression, stress responses, and cytokine release through its responses to extracellular stimuli [1]. However, both deregulated expression and uncontrolled action of TCTP are implicated in many pathological conditions, such as allergies and inflammation, tumor progression, and neurodegenerative disorders [1]. Diverse intracellular functional mechanisms of TCTP/HRF have been presented in numerous studies, although detailed mechanisms are still elusive. It has been suggested that the receptor and signaling mechanisms of TCTP/HRF might not yet have been fully identified. 

Many studies have indicated the potential association of TCTP in the physiology and pathology of the nervous system. TCTP transcript has been shown to be expressed abundantly in many areas of embryonic and adult neurons [6]. TCTP’s implications in pathophysiological conditions in the human brain was first reported by Kim et al. [7], specifically that significantly decreased TCTP expression was observed in the temporal cortices of patients with Alzheimer’s disease and Down’s syndrome. Later, Chung et al. [8] also demonstrated the reduced level of TCTP levels in the schizophrenia hippocampus. This evidence suggests that TCTP is involved in the physiological regulation of cognitive functions in the hippocampus and that decreased TCTP may result in the development of neurodegenerative or dementia-triggering disorders. An increasing number of studies further suggest the importance of TCTP in the brain and neuronal cell systems by showing that TCTP mRNAs or proteins are enriched in specific neuronal compartments, especially in the axonal region of neurons [9,10,11,12]. Based on the TCTP expression in PC12 cells and in brain regions in which dopaminergic neuronal pathways are present (Figure 1), here, we hypothesized here that TCTP might also regulate the neurotransmitter release, suggesting its novel functional role in neuronal systems. 

In neuronal cells including PC12 cells, membrane depolarization by KCl resulted in Ca^2+^ influx through depolarization-induced activation of voltage-gated Ca^2+^ channels, which thereby triggered neurotransmitter release [13,26]. Extracellular Ca^2+^ influx was also suggested to play an essential role in bradykinin receptor-mediated dopamine release from PC12 cells [27,28]. We found that dopamine release from PC12 cells was stimulated by exogenous treatment with rTCTPs, which not only triggered basal dopamine release in concentration- and time-dependent manners but also additively increased the high K^+^-stimulated (depolarization-induced) dopamine release in PC12 cells (Figure 2). These results indicate that the rTCTP-evoked dopamine release might different from that of high K^+^-stimulated exocytotic dopamine release. In addition, rTCTP evoked an evident cytosolic Ca^2+^-response but it was not related to the rTCTP-induced dopamine release in PC12 cells (Figure 3 and Figure 4). The stimulation of intracellular Ca^2+^-response and dopamine release by high K^+^ (50 mM KCl) or bradykinin was observed to be extracellular Ca^2+^-dependent (Figure 3B and Figure 4A). In contrast, RrTCTP stimulated dopamine release even in the absence of extracellular Ca^2+^ (Figure 4A) or blocking L-type Ca^2+^ channels by nifedipine (Figure 4C), although the RrTCTP-evoked increase in [Ca^2+^]_i_ was abolished in Ca^2+^-free media (Figure 3A) and media that contained BAPTA-AM (Figure 3C).

The role of Ca^2+^ in neurotransmitter release has been widely investigated, but extracellular Ca^2+^-independent release of various neurotransmitters including, acetylcholine [29], γ-aminobutyric acid [30], glutamate [31], and dopamine [32] has also been reported in depolarizedf brain slices or synaptosomes. One explanation for the extracellular Ca^2+^-independent neurotransmitter release is that depolarization induces a conformational change in certain cell membrane proteins to be sensitive to intracellular Ca^2+^, leading to stimulate exocytosis [33]. According to this hypothesis, the mobilization of Ca^2+^ from intracellular Ca^2+^ stores could be a prerequisite for neurotransmitter release. Therefore, we investigated whether modifying intracellular Ca^2+^ would affect rTCTP-stimulated dopamine release. Of interest, rTCTP-stimulated dopamine release does not need to be accompanied by changes in cytosolic Ca^2+^ because MrTCTP induced dopamine release even when the intracellular Ca^2+^ was buffered with a cell-permeant Ca^2+^ chelator in the presence of extracellular Ca^2+^ (Figure 3C and Figure 4B), as well as in the absences of extracellular Ca^2+^. It is also reasonable to assume that Ca^2+^ mobilization from intracellular stores is not the major mechanism by which rTCTP stimulates dopamine release, as evidenced by our observation that treatment of PC12 cells with dantrolene—which blocks Ca^2+^ release from the ER—did not affect MrTCTP-induced dopamine release in the presence of extracellular Ca^2+^ (Figure 4D). 

The ability of rTCTP to stimulate neurotransmitter release in the absence of extracellular Ca^2+^ and without Ca^2+^ mobilization from intracellular stores provide a unique view for the mechanisms of Ca^2+^-independent neurotransmitter release. Arachidonic acid and its metabolites are likely candidates in Ca^2+^-independent neurotransmitter release [17,34]. PLA_2_s are involved in a variety of cellular functions, including signal transduction and exocytosis [19,35]. In a number of studies with PC12 cells, PLA_2_s are suggested to be involved in both Ca^2+^-dependent exocytosis [36,37,38,39] and in Ca^2+^-independent [34] exocytosis. All major PLA_2_ groups (cPLA_2_s, sPLA_2_s, and Ca^2+^-independent iPLA_2_s) are present in the central nervous system [40,41], and iPLA_2_ was also suggested to be the major PLA_2_ in the cytosolic fraction of developing and adult rat brains [42]. We showed that rTCTP-triggered dopamine release was significantly blocked by an iPLA_2_ inhibitor, BEL, but not by inhibitor of either cPLA_2_ (AACOCF_3_) or sPLA_2_ (TEA-PC) inhibitors (Figure 5A). The iPLA_2_-dependent dopamine release by rTCTP was not accompanied with the rTCTP-evoked cytosolic Ca^2+^ response that was not affected by iPLA_2_ inhibition (Figure 5C). 

A number of studies have proposed iPLA_2_ as an essential molecular player in store-operated Ca^2+^ entry in many excitable and non-excitable cells [24,43], but this mechanisms is not likely to be involved in cytosolic Ca^2+^ response induced by rTCTP. When extracellular Ca^2+^ was removed to rule out the contribution of Ca^2+^-dependent enzymes, BEL still had its inhibitory effect on dopamine release enhanced by rTCTP. Vonakis et al. [44] reported that rTCTP/HRF increased the arachidonic acid metabolite leukotriene C_4_ from basophils, which is related to the intracellular Ca^2+^ response induced by rTCTP/HRF. In contrast, HRF/TCTP could induce secretion of both histamine and interleukin-4 in human basophils [45], in which histamine release did not need to be accompanied by changes in cytosolic Ca^2+^ [46]. From our data, it can be postulated that TCTP may be involved in the increases of arachidonic acid and its metabolites through Ca^2+^-independent PLA_2_ pathways resulting in dopamine release in neuronal cells. Although BEL is known to be a selective and potent inhibitor of iPLA_2_ (iPLA_2_β), it is also reported to inhibit non-PLA_2_ enzymes in signal transduction (e.g., phosphatidate phosphohydrolase), which may be responsible for the arachidonic acid release [47]. The involvement of signaling pathways other than iPLA_2_ cannot be ruled out in the neurotransmitter release mechanisms of TCTP through Ca^2+^-independent signaling. Our future studies aim to decipher the specified mechanisms of TCTP in dopamine release and to dissect the signaling pathways how activated PLA_2_ is linked with the process of neurotransmitter release.

## 4. Materials and Methods

### 4.1. Materials and Reagents

[^3^H]Dopamine (125 µCi/mmol) was purchased from Amersham Bioscience Corp. (Piscataway, NJ, USA) and the Scintillation cocktails (ScintiSafe^TM^ Econo2) were from Thermo Fisher Scientific (Waltham, NJ, USA). Bromoenol lactone (BEL), arachidonyl trifluoromethyl ketone (AACOCF_3_), and 1-palmitylthio-2-palmitoylamino-1,2-dideoxy-sn-glycero-3-phosphorylcholine (TEA-PC) were from Biomol Research Laboratories (Plymouth Meeting, PA, USA). Nickel-nitrilotriacetic acid (Ni-NTA) resins were from Qiagen (Stanford, CA, USA). Fura-2 acetoxymetyl ester (Fura-2-AM) was purchased from Molecular Probes (Eugene, OR, USA). Pargyline, desipramine, bradykinin, nifedipine, dantrolene, ethylene glycol-bis(β-aminoethyl ether)-*N*,*N*,*N*′,*N*′-tetraacetic acid (EGTA), 1,2-bis(2-aminophenoxy)ethane-*N*,*N*,*N*′,*N*′-tetraacetic acid acetoxymethyl ester (BAPTA-AM), isopropylthiogalactoside (IPTG), and other chemicals and reagents were purchased from Sigma Aldrich (St. Louis, MO, USA) or BD Difco (Frankline Laks, NJ, USA). Stock solutions for Fura-2-AM, BAPTA-AM, BEL, AACOCF_3_, TEA-PC, dantrolene, and bradykinin were prepared by dissolving the compounds in dimethyl sulfoxide (DMSO), and then further diluted in serum-free media; final DMSO concentrations did not exceed 0.2% (*v*/*v*). RPMI 1640, penicillin/streptomycin, horse serum, and fetal bovine serum (FBS) were obtained from GIBCO (Grand Island, NY, USA). 

### 4.2. Expression and Purification of TCTP

Rat homolog of TCTP (RTCTP) cDNA was cloned into a T7 expression vector, pRSET-A and expressed as a hexa-histidine-tagged protein. Mouse TCTP (MTCTP) cDNA was cloned into a pGEX-4T3 plasmid vector and expressed as a fusion protein with the glutathione *S*-transferase (GST). GST occurs naturally as a 26 kDa protein that can be expressed in *E. coli* with full enzymatic activity. The recombinant plasmids were transformed into *E. coli* BL21(DE3)pLysS and were expressed by the protein induction under treatment with 0.4 or 1 mM IPTG. Expressed proteins including MrTCTP and RrTCTP were purified by metal-affinity chromatography using nickel-nitrilotriacetic acid (Ni-NTA) metal-affinity chromatography and glutathione-agarose affinity chromatography, respectively, according to the manufacturer’s instructions. The contents of the purified proteins were determined with the Pierce-BCA protein assay kit (Rockford, IL, USA) using bovine serum albumin (BSA), and were analyzed by sodium dodecyl sulfate (SDS)-polyacrylamide gel electrophoresis (PAGE). Purified proteins were stored at −70 °C until use.

### 4.3. Neuronal Cell Culture

Rat PC12 cells (ATCC number: CRL-1721) were grown in RPMI 1640 media supplemented with 10% horse serum, 5% fetal bovine serum (FBS), 100 units/mL penicillin, and 100 µg/mL streptomycin. The cultures were maintained in an incubator at 37 °C in humidified conditions under 5% CO_2_. The medium was changed twice weekly, and the cultures were split at 1:5 ratios every five days. Passages 5 through 15 were used. In all experiments, the cells were plated on two-well dishes coated with poly-l-lysine (0.01 mg/mL).

### 4.4. SDS-Polyacrylamide Gel Electrophoresis (PAGE) and Western Blotting

PC 12 cells were lysed with modified radioimmunoprecipitation assay (RIPA) lysis buffer (50 mM Tris-HCl, 150 mM NaCl, 1% NP-40, 0.25% deoxycholate, 1 mM EGTA, pH 8.0), supplemented with 1% protease inhibitor cocktail was added to the lysis buffer immediately prior to use). The whole-cell lysate was prepared by 12,200× *g* for 20 min at 4 °C, and the supernatant was collected. Rat brain tissues were collected from two adult rats (6–8 weeks old) in a cold lysis buffer (1% Triton X-100, 1 mM ethylenediaminetetraacetic acid (EDTA) in phosphate buffered saline (PBS) , protease inhibitor cocktail), homogenized and put on ice for 20 min, and then centrifuged at 10,000× *g* for 10 min at 4 °C. Protein content was determined in the homogenized striatal tissue samples by using a BCATM protein assay kit (Thermo Fisher Scientific (Waltham, NJ, USA)), and was assessed for Western blotting analyses. Equal aliquots of the samples were denatured at 100 °C and then electrophoresed through 12% SDS-PAGE. Proteins were transferred to polyvinylidene difluoride (PVDF) membranes in transfer buffer (25 mM Tris-Cl, pH 8.3, 192 mM glycine, 20% methanol) for 2 h. Nonspecific bindings were blocked in Tris-buffered saline (pH 7.4) containing 0.1% tween 20 (TBS-T) and 5% BSA for 2 h at room temperature. Primary anti-TCTP antibodies (kindly provided by Prof. Kyounglim Lee at Ewha Womans University) were diluted (1:4000) in TBS-T (137 mM NaCl, 20 mM Tris-Cl, 0.1% Tween 20, pH 7.6) and incubated with the membrane for 2 h. Excess primary antibody was removed by washing the membranes four times in TTBS. The blots were then incubated with secondary antibody diluted in TBS-T (1:3000). Immunoreactive proteins were detected by enhanced chemiluminescence (ECL) system using a luminescent image analyzer LAS-1000 and IMAGE GAUSE software (Fuji Photo Film, Tokyo, Japan).

### 4.5. [^3^H]Dopamine Release

PC12 cells were grown in 24-well dishes in serum-containing medium for 24 h at 37 °C. The medium was removed and cells were loaded with fresh serum-free medium containing [^3^H]dopamine (0.5 µCi/mL) for 3 h at 37 °C. The medium was removed and the cells were washed three times with PBS containing 1 mM ascorbic acid. To measure both basal and depolarized dopamine release, cells treated with various indicated reagents including rTCTPs were incubated at 37 °C for 20 min in 1 mL Krebs-Ringer-HEPES (4-(2-hydroxyethyl)-1-piperazineethanesulfonic acid) buffer (125 mM NaCl, 5 mM KCl, 2 mM CaCl_2_, 10 mM HEPES, 1.2 mM MgSO_4_, 1.2 mM KH_2_PO_4_, 6 mM glucose, 5 mM NaHCO_3_, supplemented with pargyline or desipramine) and the buffer containing 50 mM KCl, respectively. EGTA (0.5 mM) was added to the Ca^2+^-free (without CaCl_2_) media. Samples were centrifuged for 10 min at 1000× *g* at 4 °C to remove floating cells, and the supernatant was harvested and 0.2 mL was measured for released radioactivity. For total radioactivity, the cells were washed with PBS and solubilized in 1 mL of 0.5 N NaOH, and 0.45 mL was measured for radioactivity. 

### 4.6. Measurement of Changes in [Ca^2+^]_i_

PC12 cells were suspended in a buffer (140 mM NaCl, 5 mM KCl, 1.5 mM CaCl_2_, 1 mM MgCl_2_, 10 mM HEPES, 5 mM glucose, pH 7.4) or Ca^2+^-free buffer (140 mM NaCl, 5 mM KCl, 1 mM MgCl_2_, 10 mM HEPES, 5 mM glucose, pH 7.4, and 0.5 mM EGTA) and were placed in a quartz-microcuvette in a thermostat-controlled cell-holder, and then Fura-2/AM was added to a final concentration of 5 µM and incubated for 30 min at 37 °C under continuous stirring in the dark. Supernatants containing extracellular Fura-2/AM were removed following gentle centrifugation of 0.5 mL aliquots, and cells were washed three times. They were then resuspended in 1 mL of buffer and incubated at 37 °C for 10 min prior to further centrifugation and resuspension in 4.5 mL of buffer at 37 °C. Then the fluorescence signal was recorded. [Ca^2+^]_i_ were determined by monitoring Fura-2 fluorescence in a Shimadzu RF-5301 spectrofluorometer (Shimadzu Scientific Instruments, Columbia, MD, USA) by the dual wavelength method of Grynkiewicz et al. [48]. Under these conditions, the ratios of the fluorescence are obtained at excitation wavelengths of 340 and 380 nm (emission = 510 nm). 

### 4.7. Statistical Analysis

Data were presented as mean ± S.E.M. and analyzed by SigmaStat software (Jandel Scientific, San Francisco, CA, USA). Pair-wise comparisons of rTCTP’s effects on dopamine release compared with the relative indicated controls were analyzed using the one-tailed Student’s *t*-test, considering a probability value of *p* < 0.05 or 0.01 was considered statistically significant. All results are expressed as the mean ± S.E.M.

## 5. Conclusions

We found a novel neuronal function of TCTP in stimulating dopamine release in both basal and high K^+^-depolarized conditions, independently of extracellular and cytosolic Ca^2+^, although TCTP induced an increase in [Ca^2+^]_i_ only under the Ca^2+^-containing condition. The current study strongly suggests that the rTCTP-induced neurotransmitter release from PC12 cells is mainly regulated by Ca^2+^-independent pathways where iPLA_2_ may be involved. Further studies are needed to verify the TCTP’s role in the dopamine release using the in vivo experimental system or primary cultured rat neuronal cells. 

## References

[1] Bommer U.A., Thiele B.J. (2004). The translationally controlled tumour protein (TCTP). Int. J. Biochem. Cell Biol..

[2] Bohm H., Benndorf R., Gaestel M., Gross B., Nurnberg P., Kraft R., Otto A., Bielka H. (1989). The growth-related protein P23 of the Ehrlich ascites tumor: Translational control, cloning and primary structure. Biochem. Int..

[3] Yenofsky R., Bergmann I., Brawerman G. (1982). Messenger RNA species partially in a repressed state in mouse sarcoma ascites cells. Proc. Natl. Acad. Sci. USA.

[4] MacDonald S.M., Rafnar T., Langdon J., Lichtenstein L.M. (1995). Molecular identification of an IgE-dependent histamine-releasing factor. Science.

[5] Nagano-Ito M., Ichikawa S. (2012). Biological effects of Mammalian translationally controlled tumor protein (TCTP) on cell death, proliferation, and tumorigenesis. Biochem. Res. Int..

[6] Thiele H., Berger M., Skalweit A., Thiele B.J. (2000). Expression of the gene and processed pseudogenes encoding the human and rabbit translationally controlled tumour protein (TCTP). Eur. J. Biochem..

[7] Kim S.H., Cairns N., Fountoulakisc M., Lubec G. (2001). Decreased brain histamine-releasing factor protein in patients with Down syndrome and Alzheimer’s disease. Neurosci. Lett..

[8] Chung C., Tallerico T., Seeman P. (2003). Schizophrenia hippocampus has elevated expression of chondrex glycoprotein gene. Synapse.

[9] Andreassi C., Zimmermann C., Mitter R., Fusco S., de Vita S., Saiardi A., Riccio A. (2010). An NGF-responsive element targets myo-inositol monophosphatase-1 mRNA to sympathetic neuron axons. Nat. Neurosci..

[10] Gumy L.F., Yeo G.S., Tung Y.C., Zivraj K.H., Willis D., Coppola G., Lam B.Y., Twiss J.L., Holt C.E., Fawcett J.W. (2011). Transcriptome analysis of embryonic and adult sensory axons reveals changes in mRNA repertoire localization. RNA.

[11] Roque C.G., Wong H.H., Lin J.Q., Holt C.E. (2016). Tumor protein TCTP regulates axon development in the embryonic visual system. Development.

[12] Taylor A.M., Berchtold N.C., Perreau V.M., Tu C.H., Li Jeon N., Cotman C.W. (2009). Axonal mRNA in uninjured and regenerating cortical mammalian axons. J. Neurosci..

[13] Di Virgilio F., Milani D., Leon A., Meldolesi J., Pozzan T. (1987). Voltage-dependent activation and inactivation of calcium channels in PC12 cells. Correlation with neurotransmitter release. J. Biol. Chem..

[14] Greene L.A., Rein G. (1977). Release of [^3^H]norepinephrine from a clonal line of pheochromocytoma cells (PC12) by nicotinic cholinergic stimulation. Brain Res..

[15] Kim J.H., Choi S., Jung J.E., Roh E.J., Kim H.J. (2006). Capacitative Ca^2+^ entry is involved in regulating soluble amyloid precursor protein (sAPPα) release mediated by muscarinic acetylcholine receptor activation in neuroblastoma SH-SY5Y cells. J. Neurochem..

[16] Thastrup O., Cullen P.J., Drobak B.K., Hanley M.R., Dawson A.P. (1990). Thapsigargin, a tumor promoter, discharges intracellular Ca^2+^ stores by specific inhibition of the endoplasmic reticulum Ca^2+^-ATPase. Proc. Natl. Acad. Sci. USA.

[17] Abu-Raya S., Bloch-Shilderman E., Lelkes P.I., Trembovler V., Shohami E., Gutman Y., Lazarovici P. (1999). Characterization of pardaxin-induced dopamine release from pheochromocytoma cells: Role of calcium and eicosanoids. J. Pharmacol. Exp. Ther..

[18] Lazarovici P., Lelkes P.I. (1992). Pardaxin induces exocytosis in bovine adrenal medullary chromaffin cells independent of calcium. J. Pharmacol. Exp. Ther..

[19] Goetzl E.J., An S., Smith W.L. (1995). Specificity of expression and effects of eicosanoid mediators in normal physiology and human diseases. FASEB J..

[20] Hermann P.M., Watson S.N., Wildering W.C. (2014). Phospholipase A_2_—Nexus of aging, oxidative stress, neuronal excitability, and functional decline of the aging nervous system? Insights from a snail model system of neuronal aging and age-associated memory impairment. Front. Genet..

[21] Balsinde J., Balboa M.A., Insel P.A., Dennis E.A. (1999). Regulation and inhibition of phospholipase A_2_. Annu. Rev. Pharmacol. Toxicol..

[22] Ramanadham S., Ali T., Ashley J.W., Bone R.N., Hancock W.D., Lei X. (2015). Calcium-independent phospholipases A_2_ and their roles in biological processes and diseases. J. Lipid Res..

[23] Murakami M., Kudo I. (2002). Phospholipase A_2_. J. Biochem..

[24] Singaravelu K., Lohr C., Deitmer J.W. (2006). Regulation of store-operated calcium entry by calcium-independent phospholipase A_2_ in rat cerebellar astrocytes. J. Neurosci..

[25] Vaudry D., Stork P.J., Lazarovici P., Eiden L.E. (2002). Signaling pathways for PC12 cell differentiation: Making the right connections. Science.

[26] Yermolaieva O., Brot N., Weissbach H., Heinemann S.H., Hoshi T. (2000). Reactive oxygen species and nitric oxide mediate plasticity of neuronal calcium signaling. Proc. Natl. Acad. Sci. USA.

[27] Fasolato C., Pandiella A., Meldolesi J., Pozzan T. (1988). Generation of inositol phosphates, cytosolic Ca^2+^, and ionic fluxes in PC12 cells treated with bradykinin. J. Biol. Chem..

[28] Weiss C., Atlas D. (1991). The bradykinin receptor—A putative receptor-operated channel in PC12 cells: Studies of neurotransmitter release and inositol phosphate accumulation. Brain Res..

[29] Adam-Vizi V., Ligeti E. (1984). Release of acetylcholine from rat brain synaptosomes by various agents in the absence of external calcium ions. J. Physiol..

[30] Schwartz E.A. (1987). Depolarization without calcium can release γ-aminobutyric acid from a retinal neuron. Science.

[31] Nicholls D.G., Sihra T.S., Sanchez-Prieto J. (1987). Calcium-dependent and -independent release of glutamate from synaptosomes monitored by continuous fluorometry. J. Neurochem..

[32] Lonart G., Zigmond M.J. (1991). High glutamate concentrations evoke Ca(++)-independent dopamine release from striatal slices: A possible role of reverse dopamine transport. J. Pharmacol. Exp. Ther..

[33] Hochner B., Parnas H., Parnas I. (1989). Membrane depolarization evokes neurotransmitter release in the absence of calcium entry. Nature.

[34] Bloch-Shilderman E., Abu-Raya S., Trembovler V., Boschwitz H., Gruzman A., Linial M., Lazarovici P. (2002). Pardaxin stimulation of phospholipases A_2_ and their involvement in exocytosis in PC-12 cells. J. Pharmacol. Exp. Ther..

[35] Bonventre J.V. (1997). Roles of phospholipases A_2_ in brain cell and tissue injury associated with ischemia and excitotoxicity. J. Lipid Mediat. Cell Signal..

[36] Matsuzawa A., Murakami M., Atsumi G., Imai K., Prados P., Inoue K., Kudo I. (1996). Release of secretory phospholipase A_2_ from rat neuronal cells and its possible function in the regulation of catecholamine secretion. Biochem. J..

[37] Ray P., Berman J.D., Middleton W., Brendle J. (1993). Botulinum toxin inhibits arachidonic acid release associated with acetylcholine release from PC12 cells. J. Biol. Chem..

[38] Song H.S., Ko M.S., Jo Y.S., Whang W.K., Sim S.S. (2015). Inhibitory effect of acteoside on melittin-induced catecholamine exocytosis through inhibition of Ca^2+^-dependent phospholipase A_2_ and extracellular Ca^2+^ influx in PC12 cells. Arch. Pharm. Res..

[39] Yang C.W., Rathinavelu A., Borowitz J.L., Isom G.E. (1994). Activation of a calcium- and pH-dependent phospholipase A_2_ by cyanide in PC12 cells. Toxicol. Appl. Pharmacol..

[40] Dennis E.A., Cao J., Hsu Y.H., Magrioti V., Kokotos G. (2011). Phospholipase A_2_ enzymes: Physical structure, biological function, disease implication, chemical inhibition, and therapeutic intervention. Chem. Rev..

[41] Farooqui A.A., Ong W.Y., Horrocks L.A. (2006). Inhibitors of brain phospholipase A_2_ activity: Their neuropharmacological effects and therapeutic importance for the treatment of neurologic disorders. Pharmacol. Rev..

[42] Yang H.C., Mosior M., Ni B., Dennis E.A. (1999). Regional distribution, ontogeny, purification, and characterization of the Ca^2+^-independent phospholipase A_2_ from rat brain. J. Neurochem..

[43] Smani T., Dominguez-Rodriguez A., Callejo-Garcia P., Rosado J.A., Avila-Medina J. (2016). Phospholipase A_2_ as a Molecular Determinant of Store-Operated Calcium Entry. Adv. Exp. Med. Biol..

[44] Vonakis B.M., Macglashan D.W., Vilarino N., Langdon J.M., Scott R.S., MacDonald S.M. (2008). Distinct characteristics of signal transduction events by histamine-releasing factor/translationally controlled tumor protein (HRF/TCTP)-induced priming and activation of human basophils. Blood.

[45] Schroeder J.T., Lichtenstein L.M., MacDonald S.M. (1996). An immunoglobulin E-dependent recombinant histamine-releasing factor induces interleukin-4 secretion from human basophils. J. Exp. Med..

[46] MacGlashan D., Botana L.M. (1993). Biphasic Ca^2+^ responses in human basophils. Evidence that the initial transient elevation associated with the mobilization of intracellular calcium is an insufficient signal for degranulation. J. Immunol..

[47] Balboa M.A., Balsinde J., Dennis E.A. (1998). Involvement of phosphatidate phosphohydrolase in arachidonic acid mobilization in human amnionic WISH cells. J. Biol. Chem..

[48] Grynkiewicz G., Poenie M., Tsien R.Y. (1985). A new generation of Ca^2+^ indicators with greatly improved fluorescence properties. J. Biol. Chem..

